# Multi-Objective Optimization of Biodegradable and Recyclable Composite PLA/PHA Parts

**DOI:** 10.3390/polym17152147

**Published:** 2025-08-06

**Authors:** Burak Kisin, Mehmet Kivanc Turan, Fatih Karpat

**Affiliations:** Department of Mechanical Engineering, Bursa Uludağ University, Bursa 16059, Turkey; burakkisin@uludag.edu.tr (B.K.); karpat@uludag.edu.tr (F.K.)

**Keywords:** pla, pha, additive manufacturing, tensile, compression, optimization, grey relational analysis

## Abstract

Additive manufacturing (AM) techniques, especially fused deposition modeling (FDM), offer significant advantages in terms of cost, material efficiency, and design flexibility. In this study, the mechanical performance of biodegradable PLA/PHA composite samples produced via FDM was optimized by evaluating the influence of key printing parameters—layer height, printing orientation, and printing speed—on both the tensile and compressive strength. A full factorial design (3 × 3 × 3) was employed, and all of the samples were triplicated to ensure the consistency of the results. Grey relational analysis (GRA) was used as a multi-objective optimization method to determine the optimal parameter combinations. An analysis of variance (ANOVA) was also conducted to assess the statistical significance of each parameter. The ANOVA results revealed that printing orientation is the most significant parameter for both tensile and compression strength. The optimal parameter combination for maximizing mechanical properties was a layer height of 0.1 mm, an X printing orientation, and a printing speed of 50 mm/s. This study demonstrates the effectiveness of GRA in optimizing the mechanical properties of biodegradable composites and provides practical guidelines to produce environmentally sustainable polymer parts.

## 1. Introduction

Fused filament fabrication (FFF) or fused deposition modeling (FDM) techniques, also known as material extrusion methods, comprise one of the popular sub-branches of the additive manufacturing method [1]. In a simple manner, this method relies on melting and cooling in a cycle of materials while controlling the deposition location in space. This method opens up new opportunities regarding the usage of materials like polylactic acid (PLA) in many ways. PLA is becoming popular as a production material with improved FDM technology. The reason for the popularity of this material is its ability to degrade in nature, its low toxicity, its high compatibility with living tissues, and its reusability; for these reasons, this material is used in various areas, such as in automotive applications [2,3,4], food packaging [5,6], medical applications [7,8,9,10], and live environment research [11]. However, PLA’s mechanical properties depend on many factors; for instance, printing parameters [1,12,13] and filament manufacturers [14]. Turan et al. [1] carried out one of the most comprehensive mechanical studies regarding PLA parameter optimization. They observed that the most effective parameter was the raster angle with respect to the tensile strength. Kafshgar et al. [12] examined the effect of printing parameters on the mechanical properties of PLA. They saw that the tensile strength increased in the filament direction and decreased with an increasing layer height. Another similar study was conducted by Gunay et al. [13]; they observed that tensile strength limited change with an increasing printing speed and increased in the filament direction. Despite the many advantages mentioned before, PLA shows poor mechanical performance in some scenarios. To overcome this situation and to widen its usage, PLA was mixed with materials like polyhydroxyalkanoate (PHA) [11], wood fiber [15], glass fiber [16], carbon fiber [17], ceramic [18], etc., and composites were created. There are many articles in the literature focused on PLA/PHA. In the study conducted by Guessasma et al. [19], the mechanical properties of PLA/PHA composite filaments with wood inserts due to manufacturing with different additive manufacturing parameters were investigated experimentally and numerically. The printing temperature was selected as a parameter in the additive manufacturing process, and printing was performed at five different temperatures. In addition to the experimental investigation of the results, the three-dimensional microstructure of the samples after printing was examined by X-ray tomography, and the mechanical properties were verified by a simulation study. One of the results revealed that above 230 °C, wood-doped materials are not suitable for printing. The other conclusion is that mechanical improvement provided by the wood additive is optimally achieved at 220 °C. PHA is a promising material to improve PLA’s poor mechanical performance without damaging its degradability, low toxicity, or compatibility with living tissue. This mechanical improvement added to the PLA, the material’s usability area, and mechanically durable scenarios. Montalvao et al. [20] conducted a study on the mechanical characteristics of PLA and PLA/PHA materials in ocean conditions as innovative material types that can be used in ocean research without polluting the ocean due to their biodegradability capabilities. Zgodavova et al. [21] conducted a study in which a face mask used for medical purposes, which is planned to be produced by additive manufacturing, was produced with different materials. The main purpose of their study was to determine a material that can be used for the production of these masks with additive manufacturing over a short amount of time, with high geometric accuracy, and with minimal waste after use. In addition to material variation, the effects of the parameters used in additive manufacturing, such as layer height, the number of walls, nozzle diameter, filling ratio, and printing temperature, were also investigated. As a result, the mechanical properties, geometric accuracy, weight, printing time, cost, and environmental impacts were analyzed. Considering all of these parameters, PHA was found to be the most suitable material for this purpose. In his study [7], Cecen examined the mechanical properties and usability of the infill patterns, which are the parameters used in additive manufacturing in the production of PLA/PHA blend material with additive manufacturing, whose performance is evaluated for tissue production in the medical field. In Zaharia et al.’s article [22], the mechanical properties of PLA/PHA, a biodegradable composite material for use in the aerospace industry, were investigated by additive manufacturing with different infill geometries. In their study, the mechanical effects of three different infill patterns, namely honeycomb, diamond, and zigzag, were studied. The mechanical properties were determined using compression, tensile, and three-point bending tests. Defects in the specimens produced with different core patterns were evaluated in terms of surface wrinkling, surface folding, and surface/core separation. In addition to the experimental setup, this study was also carried out on the numerical plane. As a result, in compression and three-point bending tests, the diamond-patterned core showed the best performance, while the zigzag core showed the best tensile performance. However, the main challenge in adding PHA to PLA and creating a composite material to enhance mechanical performance is to determine critical parameters to achieve optimal performance, namely the mixing ratio of PLA/PHA and printing parameters of FDM. In the literature, various studies have been conducted to reach the optimal mixing ratio and printing parameters. In the article conducted by Pop et al. [23], Acrylonitrile Butadiene Styrene (ABS), PLA, and bamboo filled PLA/PHA-type materials were manufactured with different additive manufacturing infill patterns, and the differences between their mechanical properties were compared. Tensile strength, three-point bending, compression strength, and crash tests were applied to the specimens. The microscope examinations showed that a 45% reduction by mass was obtained with the standard square infill pattern and 60% with the tube infill pattern. Subsequently, the authors proposed a fully filled ABS configuration for tensile strength, a fully filled PLA configuration for compression strength, a fully filled PLA configuration for flexural strength, and a PLA/PHA configuration with bamboo filling with the tube infill pattern for crash resistance. Chatrath et al. [24] investigated the recyclability of PLA/aPHA blends at 75% and 90% by weight compared with PLA alone. The materials were subjected to five different heat-history processes, and the resulting mechanical, flow, and thermal property changes were investigated. It was stated that the tensile properties did not change during the reprocessing process, while the isode collision resistance of PLA decreased by 75%. The authors reported that the inclusion of 25 wt% aPHA improved the extrusion ability, elongation fracture, and collision resistance, with the authors suggesting that it would be useful for thermoforming packaging. Mondragon-herrera et al. [25] examined and compared the mechanical properties of PHA and PLA and the material obtained from the mixture of these two polymers in certain ratios. It was determined that PLA had high hardness and tensile strength but low toughness. PHA alone has low mechanical performance, but when added to PLA as an additional material, the toughness was much higher than that of PLA alone. The authors also reported that the PLA/PHA blend performed much better than the other materials in terms of thermal stability. Finally, it is emphasized that the material consisting of PLA/PHA blend reduces the cold crystallization and glass transition temperature, which is beneficial in the additive manufacturing process. Aldam et al. [26] investigated the mechanical properties of PLA, PHA, and a blend of the two in different ratios. The material consisting of the PLA/PHA blend had lower tensile strength compared with pure PLA. The tensile strength decreased gradually as the ratio of PHA added to PLA increased, and the highest tensile strength was seen with a 50–50 ratio. PHA had lower tensile strength than pure PLA and PLA/PHA blends. Guessasma et al. [27] experimentally and numerically investigated the parametric variations effect of PLA/PHA blend material for an additive manufacturing process. Printing temperature was selected as one of the additive manufacturing parameters in the study. Tensile tests were performed on specimens printed with six different values of this parameter, and numerical analyses were carried out. As a result, it is emphasized that the variation in the printing temperature has a great effect on the brittleness. It was stated that the high cooling rates in printing processes at high temperatures made the specimens more brittle than printing at low temperatures. Another output is that the printing temperature is related to the orientation of the cracks formed on the sample. While there is no specific orientation of crack progression at low printing temperatures, it was noted that cracks progress in the direction of the raster angle at high temperatures. Oviedo et al. [28] experimentally investigated the mechanical property changes in PLA and PLA/PHA blend materials depending on the additive manufacturing parameters. The raster angle was determined as a research parameter, and three different raster angle values were selected. The samples were evaluated for their tensile, bending, impact, and fragmentation performances. It was observed that the effect of the raster angle on the tensile and bending values was weak. Efstathiadis et al. [11] investigated the mechanical property performances of the material obtained from pure PLA and PLA/PHA mixtures on the samples produced by the additive manufacturing method. This study aimed to reconstruct the skeleton of a sea creature using an additive manufacturing method. One of the results showed that the tensile strength of pure PLA material was significantly higher than PLA/PHA–wood material and significantly higher than PLA/PHA material. Ali et al. [29] investigated the PLA/PHA blend material produced by an additive manufacturing method with different additive manufacturing parameters. Three different printing parameters and three different levels of these parameters were selected. The selected parameters were layer height, printing temperature, and flow rate. It was stated that layer height and flow rate had a noticeable effect on the mechanical properties.

In this study, the mechanical performance of PLA/PHA composite material that depends on the FDM printing parameters was experimentally investigated. Three printing parameters were chosen: layer height, printing speed, and printing orientation. These parameters had three sub-levels. A full factorial design of the experiment was conducted to see all of the parametrical changes clearly. Two different outputs were planned to be observed: tensile and compression strength. In addition to addressing the gaps identified in the literature, this study aimed to simultaneously determine the optimal combination of printing parameters that maximize both tensile and compressive strength and analyze their respective effects on the mechanical performance of PLA/PHA composites.

## 2. Materials and Methods

**Figure 1:**
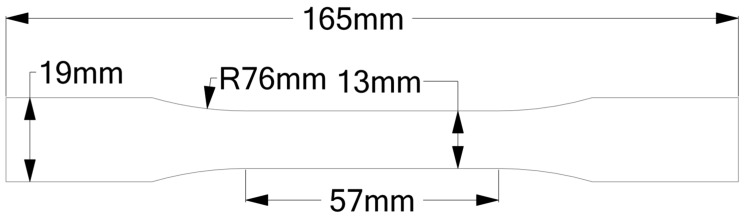
ASTM D638 [35] type 1 tensile sample sizes.

**Figure 2:**
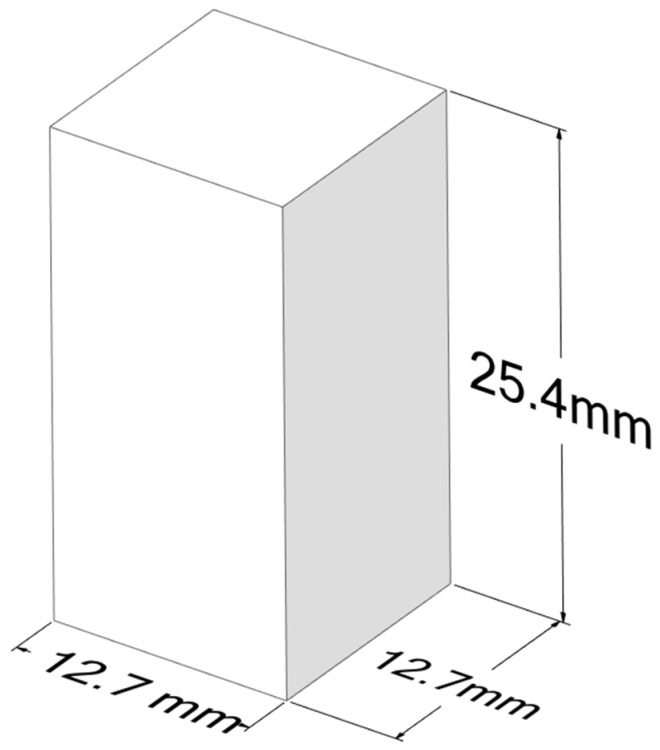
ASTM D695 [36] compression sample sizes.

In this study, the SOLVIX brand of PLA/PHA composite filament was used as a printing material. The filament contains approximately 28% PHA. The most significant advantage that PHA provides is the increased flexibility of PLA. Many articles in the literature contain different proportions of PHA, especially high amounts of PHA [24,26,30,31,32]. General information about the material properties of PLA and PHA is given in Table 1 [33,34]. Two different types of mechanical tests were used: tensile and compression tests. American Society for Testing and Materials (ASTM) D638 [35] type 1 sizes were used for the tensile tests [1] with 3 mm thickness, and ASTM D695 [36] sizes were used for the compression tests [37]. Ansys Workbench Spaceclaim software (2022 R1) was used for the computer-aided design (CAD) of the specimens; the sizes are given in Figure 1 and Figure 2.

A full factorial experimental design was used to see the parameter effects clearly. Three samples were produced and tested for each experimental set. The parameters and parameters’ levels are given in Table 2. When the literature was investigated, it was seen that printing speed, printing temperature, layer height, raster angle or printing orientation, infill pattern, infill density, and heated-bed temperature were generally studied in additive manufacturing studies. However, since both compressive strength and tensile strength aimed to be optimized together in this study, infill density was taken as 100%, and therefore infill pattern was not a research parameter. In addition, since the research material is a composite material, preliminary studies were carried out for heated-bed temperature and printing temperature to ensure the consistent production of printing samples and that appropriate parameters were subsequently determined. It is suggested that in the literature [38] that the maximum layer height should be 75% of nozzle diameter, which is 0.4 mm for this study. Therefore, the maximum layer height in this study can be 0.3 mm. The minimum suggested layer height for the 3D printers used in this study is 0.1 mm; therefore, the minimum layer height in this study can be 0.1 mm. Based on this information, layer heights were selected as the maximum possible value of 0.3 mm, the minimum possible value of 0.1 mm, and the mid value of the maximum and minimum value of 0.2 mm. Maximum printing speed tests were performed for the Z-orientation and 0.3 mm layer height, which were predicted to be the most difficult dimensional consistencies to achieve, and 60 mm/s was detected as the ideal speed. In order to not extend the printing time too much, another two printing speeds of 40 mm/s and 50 mm/s were also selected.

**Figure 3:**
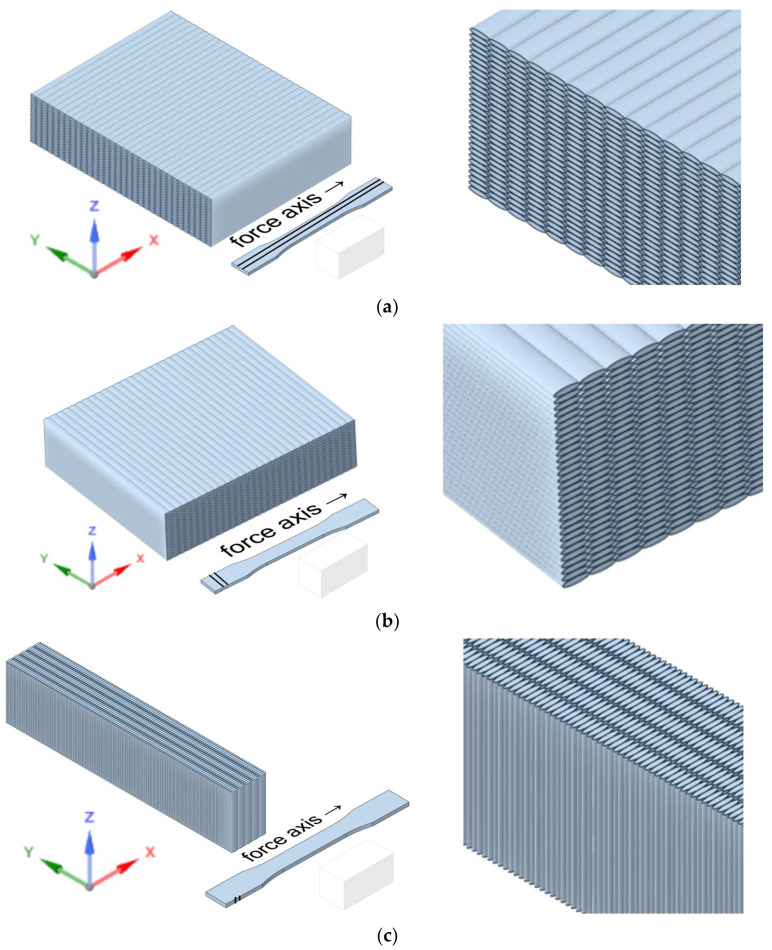
Printing orientations: (**a**) X-orientation; (**b**) Y-orientation; (**c**) Z-orientation.

Printing orientations are shown in Figure 3.

Creality Slicer 4.8.2 was used as the slicer software; this software was developed by Shenzhen Creality 3D Technology Co., Ltd, Shenzhen, China. The heated-bed temperature was 50 °C, and the printing temperature was 230 °C. These values were selected based on filament manufacturer recommendations and printing tries by the authors. In addition, printing errors were encountered at different temperatures. A full factorial experimental design was used; for this reason, the Creality ender 3 v3 se 3D (Manufacturer: Shenzhen Creality 3D Technology Co., Ltd., Shenzhen, China) printer (0.4 mm diameter nozzle) was used to manufacture the tensile samples, and the Creality cr-10 se 3D (Manufacturer: Shenzhen Creality 3D Technology Co., Ltd., Shenzhen, China) printer (0.4 mm diameter nozzle) was used to manufacture the compression samples. Thus, printing time was reduced. The KAL-MET universal test (Manufacturer: KAL-MET Kalibrasyon Trade Co. Ltd., Bursa, Turkey) machine was used for the tensile tests because this machine has a 10 kN load cell, and the ultimate tensile values were anticipated to be lower due to the cross-sectional area. All tensile tests were conducted at 10 mm min^−1^.

On the other hand, the UTEST universal test (Manufacturer: UTEST Malzeme Test Cihazları ve Makina İmalatı and foreign trade Inc., Ankara, Turkey) machine was used for compression tests because this machine has a 200 kN load cell. All of the compression tests were conducted at 5 mm min^−1^.

**Figure 4:**
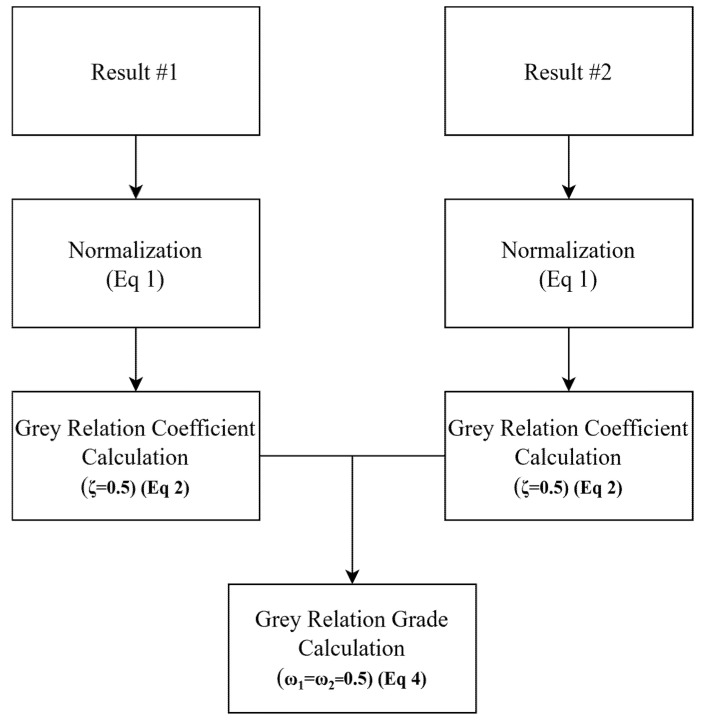
Grey relational analysis flowchart.

This study had two objectives: to maximize tensile strength and compression strength via the optimization of printing parameters. Multi-objective grey relational analysis was considered a suitable method for this study (Figure 4). This method has been used in many additive manufacturing articles [18,39].

Multi-objective optimization aims to optimize two or more factors, and these factors have different units. For this reason, first of all, the factors must be normalized to purge from their units. Depending on the study, the objective is to maximize or minimize the value. Equation (1) was used in this study since both objectives were to maximize [40,41].

(1) $$y_{i}(k)=\frac{x_{i}^{0}\left(k\right)-min\;x_{i}^{0}\left(k\right)}{max\;x_{i}^{0}\left(k\right)-min\;x_{i}^{0}\left(k\right)}$$

In this equation, $x_{i}^{0}\left(k\right)$ means any value of any factor, $max\;x_{i}^{0}\left(k\right)$ means the maximum value of $x_{i}^{0}\left(k\right)$, $min\;x_{i}^{0}\left(k\right)$ means the minimum value of $x_{i}^{0}\left(k\right)$, and $y_{i}(k)$ means the normalized value [40,41]. After this process, the grey relational coefficient (GRC) is calculated; for this purposes, Equation (2) is used [40,41].

(2) $$y_{i}(k)=\frac{∆min+\zeta ∆max}{{∆}_{0i}(k)+\zeta ∆max}$$

(3) $${∆}_{0i}\left(k\right)=\left|y_{0}\left(k\right)-y_{i}(k)\right|$$

In Equation (2), ζ means the discrimination coefficient; the default value of the discrimination coefficient is 0.5, which was used in this study [40]. ∆max means the maximum value of ${∆}_{0i}$ and ∆min means the minimum value of ${∆}_{0i}(k)$ means the difference between $y_{0}\left(k\right)$ and $y_{i}(k)$ [40,41]. At this point, $y_{0}\left(k\right)$ means the comparability sequence, and $y_{i}(k)$ means the reference sequence [40,41].

Finally, the Grey Relational Grade (GRG) must be calculated; for this purpose, Equation (4) was used [40,41].

(4) $$GRG=\sum_{k=1}^{n}\omega_{k}y_{i}\left(k\right)$$

(5) $$\sum_{k=1}^{n}\omega_{k}=1$$

In Equations (4) and (5), $\omega_{k}$ means the normalized weight factor of each response [40,41].

**Figure 5:**
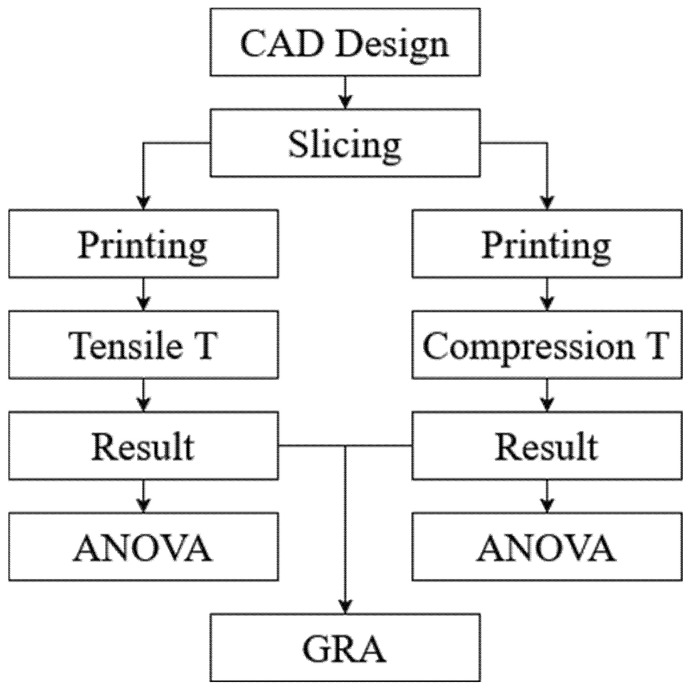
Flowchart of the study.

An analysis of variance (ANOVA) was used to determine the contribution ratio and rank of the investigation parameters and detect the accuracy of the proposed hypothesis. The flowchart of this study is shown in Figure 5.

## 3. Results and Discussion

First of all, the tensile and compression test results were examined.

### 3.1. Test Results

**Figure 6:**
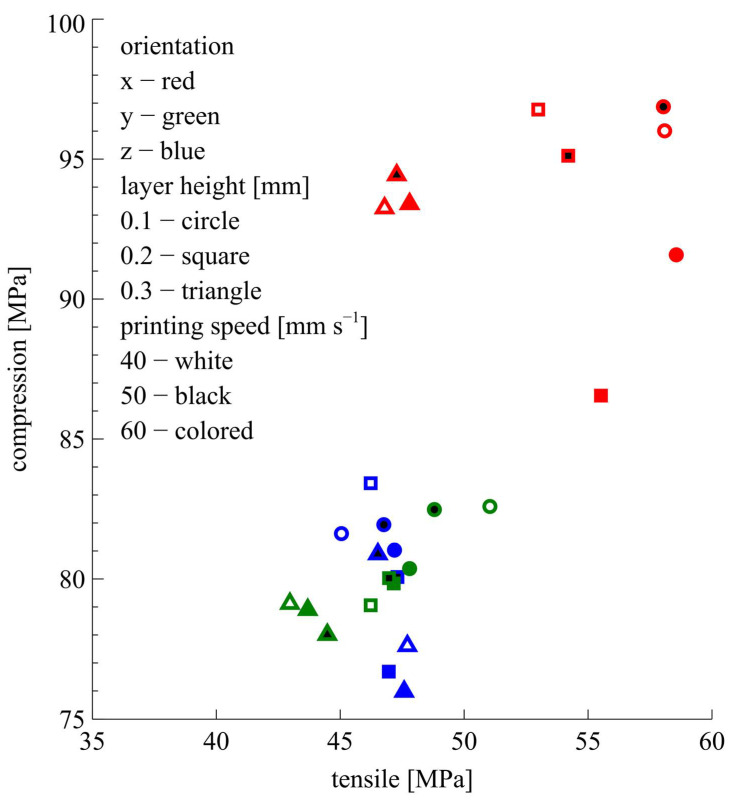
Tensile and compression test results.

The means of the tensile test results and the means of the compression test results are given in Figure 6 and Table 3.

As can be seen in Table 3 and Figure 6, printing orientation and layer height have an obvious effect on the tensile and compression strengths. However, to see the effect of these parameters more clearly, the averages of the results need to be examined. In this context, the change in the means of the results was examined; also, an ANOVA was performed. First of all, the tensile test results were examined.

**Figure 7:**
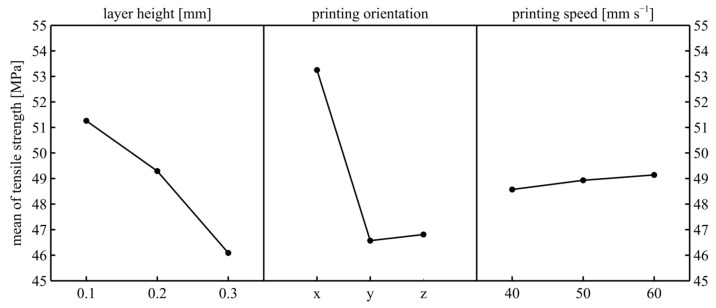
Mean of tensile strength.

When Figure 7 was examined, it was seen that the optimal printing parameter set for the maximum tensile strength was 0.1 mm layer height, X printing orientation, and 60 mm/s printing speed. When the changes in the means were examined, the effects of layer height and printing orientation were clearly seen. These results are compatible with those in the literature [12].

The main reason the X printing orientation has the highest strength is that this orientation shows filament strength. However, the primary reason both Y- and Z-orientations show less strength is the examination of the strength of the filament adhesion in these orientations. The small difference between these two results is that the number of filaments adhering in the Z-orientation is higher.

When the layer height results were examined, it was observed that the strength decreased as the layer height increased. The main reason for this situation is the decrease in the number of filaments subject to tension. The strength changed slightly with printing speed. The level of change in the tensile strength that was dependent on printing speed is consistent with Gunay’s study [13]. This is because a narrow range of printing speed must be selected since the filament was unsuitable for high speeds. In addition, a low printing speed would increase the manufacturing time considerably. Some of the existing printing sets have a printing time of over four hours. This situation prevented the printing speed from being reduced too much. Additionally, this study was carried out with a full factorial design, and the samples were printed one sample at a time to ensure printing consistency. The reason for the increase in strength as the printing speed increases could be that the temperature difference between the two consecutively printed layers was lower as the printing speed increases; therefore, the adhesion was better. 

When the ANOVA results were examined (Table 4), the *p*-value values clearly showed that layer height and printing orientation were very effective parameters in terms of tensile strength. However, printing speed had no statistically significant effect. When the contribution rates were examined, the highest contribution rate belongs to the printing orientation, with 51.25%. This result is consistent with Turan’s study [1]. When the interaction effect results were examined, it was seen that the interaction effect of layer height × printing orientation was statistically significant. Since these two parameters were statistically effective independently, they are expected to be effective as an interaction effect.

At the next stage, the compression results were examined.

**Figure 8:**
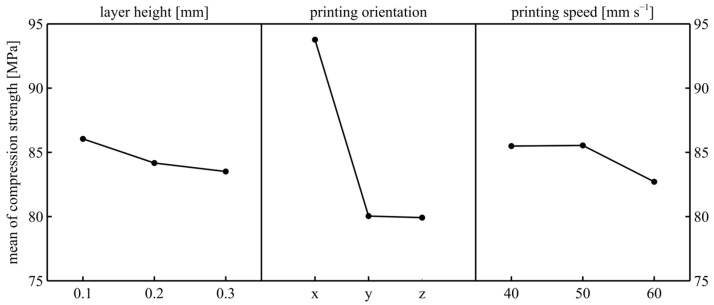
Mean of compression strength.

When Figure 8 was examined, it was seen that the optimal printing parameter set for the maximum compressive strength was 0.1 mm layer height, X printing orientation, and 50 mm/s printing speed. When the changes in the means were examined, the dominance of the printing orientation was clearly observed. The main factor of the X printing orientation has the highest strength because the filaments are subjected to buckling in this orientation, while the Y- and Z-orientations show less strength because of the strength of the filament adhesion observed in these orientations. The reason for the results in these Y- and Z-orientations is that the samples are a square section and the Y- and Z-orientations do not differ with respect to the printing axis. When the layer height results were examined, it was observed that the compressive strength decreased as the layer height increased; this is compatible with Saravanamuthukumar’s work [42], but the rate of change was quite low. The reason for this is the decrease in the number of filaments subjected to compression. The strength decreased as the printing speed increased to 60 mm/s; this result is also compatible with Saravanamuthukumar’s work [42]. The reason for this is expected to be the decrease in production quality. When the ANOVA results were examined (Table 5), the *p*-value clearly showed that printing orientation and printing speed were statistically effective on the compressive strength. Layer height can be considered statistically effective since the *p*-value was very close to 0.05. In addition, the interaction effects appeared to be ineffective. The printing speed was statistically ineffective on tensile strength but statistically effective on compressive strength because the printing speed affects the dimensional consistency of the samples. Accordingly, while the ratio of the standard deviation measured depending on the printing speed to the average sample cross-sectional area in the samples printed for tensile tests was below 0.50%, the ratio of the standard deviation measured depending on the speed to the average sample cross-sectional area in the samples printed for compression tests was above 1.25%. The values were calculated as follows: 1—Calculate the mean value of compression and tensile under the same printing speed separately. 2—Find the standard deviation of the compression and tensile samples under different speeds separately. 3—Find the calculation ratio of the standard deviations and mean values. Thus, it is obvious that the printing speed will be more effective in compressive samples. When the contribution rates are examined, the highest contribution rate belongs to the printing orientation with 88%. The reasons for the low error rate are the dominant effect of the printing orientation, the production of the printing samples from a single coil of filament, and the pure geometry of the printing sample.

### 3.2. Grey Relational Analysis Results

As mentioned before, one of the objectives of this study was to determine the right combination of the input parameters that simultaneously gives the maximum value of the compression and tensile strength. The grey relational analysis (GRA) method was used to do this. Table 6 shows the results of the GRA. According to Table 6, different samples had maximum and minimum compression and tensile strength values.

The input combination of 0.10 mm layer height, X-orientation, and 60 mm/s (i.e., sample no. 3) had the highest tensile strength with a 1.0 tensile strength coefficient value in GRA. Also, 0.30 mm layer height, Y-orientation, and 40 mm/s (i.e., sample no. 22) had the lowest tensile strength with a 0.33 tensile strength coefficient. The reasons for these results were discussed before in Section 3.1.

The input combination of 0.10 mm layer height, X-orientation, and 50 mm/s (i.e., sample no. 2) had the highest compression strength with a 1.0 compression strength coefficient value in GRA. Also, 0.30 mm layer height, Z-orientation, and 60 mm/s (i.e., sample no. 27) had the lowest compression strength with a 0.33 compression strength coefficient. The reasons for these results were discussed before in Section 3.1.

The combination that gave the highest tensile strength was the eighth highest value for the compression tests. Additionally, the combination that gave the highest compression strength was the third highest value for the tensile tests. One possible reason for this result is that tensile and compression strength are affected inversely from printing speed. As mentioned before, while the ascension of printing speed reduced the compression strength, it increased the tensile strength. Additionally, the parameter set with the highest tensile strength ranks eighth in terms of compression, and one of the highest was the other’s third, because tensile and compression have affected printing speed to different degrees. In Section 3.1, the *p*-values are presented. For tensile strength, the *p*-value of the printing speed was 0.514, and for compression strength, the *p*-value was 0.027. These results mean, statistically, that tensile strength is not affected by printing speed, while compression strength is affected.

Within all these results, the best combination for combined strength was sample no. 2 with input parameters of 0.10 mm layer height, X-orientation, and 50 mm/s printing speed with a GRA score of 0.97.

## 4. Conclusions

Additive manufacturing helps save time and cost for low-volume production or prototype production. Although there are many additive manufacturing techniques, the use of the FDM method in particular is becoming more widespread day by day. PLA is the most used material in this method, but its mechanical, visual, etc., properties sometimes cannot meet the user’s requirements. For this reason, PLA is reinforced with additional materials; thus, composite PLA materials are created. In this study, PLA/PHA biodegradable composite filament was used as the investigation material. Products fabricated with additive manufacturing are greatly affected by printing parameters in terms of mechanical, visual, thermal, etc., properties. Therefore, determining the optimal parameters is of great importance. Many products are exposed to different load cases; for this reason, determining optimal printing parameters for only one load case is not enough. This study applied a multi-objective optimization for the most common loading types: tensile and compression. As a result of the optimization, printing orientation X, layer height 0.1 mm, and printing speed 50 mm/s were found as optimal printing parameters. In addition, the ANOVA determined printing orientation as the most effective printing parameter for tensile strength and compression strength. Future works will focus on optimizing the thermal properties.

## Figures and Tables

**Table 1:** Material properties of PLA and PHA.

Material	Density (g/cm^3^)	Melt Flow Index (g/10 min)
PLA	1–1.5	4–22
PHA	1.25	15–30

**Table 2:** Printing parameters and levels.

Printing Parameters	Level 1	Level 2	Level 3
Printing Orientation	X	Y	Z
Printing Speed	40 mm/s	50 mm/s	60 mm/s
Layer Height	0.1 mm	0.2 mm	0.3 mm

**Table 3:** Experimental results.

Experimental No	Layer Height [mm]	Printing Orientation	Printing Speed [mm/s]	Tensile Strength [MPa]	Tensile Strength Upper Value [MPa]	Tensile Strength Lower Value [MPa]	Compression Strength [MPa]	Compression Strength Upper Value [MPa]	Compression Strength Lower Value [MPa]
1	0.1	*X-*axis	40	58.09	58.51	57.30	96.01	97.70	94.46
2	0.1	*X-*axis	50	58.04	60.91	55.78	96.87	97.45	96.39
3	0.1	*X-*axis	60	58.56	61.49	57.07	91.58	95.61	88.58
4	0.1	*Y-*axis	40	51.04	51.81	50.28	82.59	85.35	78.13
5	0.1	*Y-*axis	50	48.80	49.32	47.79	82.48	83.95	80.56
6	0.1	*Y-*axis	60	47.80	50.04	45.01	80.37	81.06	79.60
7	0.1	*Z-*axis	40	45.05	45.87	44.24	81.62	84.75	77.75
8	0.1	*Z-*axis	50	46.76	47.60	37.86	81.94	84.36	80.67
9	0.1	*Z-*axis	60	47.19	49.77	41.94	81.03	84.29	75.78
10	0.2	*X-*axis	40	52.99	55.80	51.02	96.77	99.42	93.24
11	0.2	*X-*axis	50	54.20	54.88	52.89	95.12	98.83	91.72
12	0.2	*X-*axis	60	55.52	56.23	54.53	86.55	97.32	70.27
13	0.2	*Y-*axis	40	46.23	46.58	45.72	79.06	84.74	75.42
14	0.2	*Y-*axis	50	46.97	47.58	46.44	80.03	86.85	76.17
15	0.2	*Y-*axis	60	47.16	47.86	46.76	79.84	84.08	76.32
16	0.2	*Z-*axis	40	46.23	47.65	44.44	83.42	85.46	82.04
17	0.2	*Z-*axis	50	47.31	48.01	46.68	80.07	82.74	75.81
18	0.2	*Z-*axis	60	46.96	48.81	44.76	76.69	84.20	62.59
19	0.3	*X-*axis	40	46.79	47.97	44.74	93.25	94.43	91.23
20	0.3	*X-*axis	50	47.28	47.98	46.13	94.43	96.11	93.02
21	0.3	*X-*axis	60	47.80	49.12	46.64	93.40	95.78	89.50
22	0.3	*Y-*axis	40	42.97	44.16	41.83	79.12	83.34	76.94
23	0.3	*Y-*axis	50	44.48	44.84	44.02	78.01	81.68	75.28
24	0.3	*Y-*axis	60	43.69	44.76	42.79	78.90	82.40	76.99
25	0.3	*Z-*axis	40	47.71	48.26	46.84	77.61	79.84	75.29
26	0.3	*Z-*axis	50	46.52	48.25	44.83	80.89	84.18	78.26
27	0.3	*Z-*axis	60	47.58	48.81	46.70	75.98	78.19	73.95

The standard deviations for the tensile and compression test results were lower than 3.

**Table 4:** ANOVA for tensile test.

Source	DF	ADJ SS	ADJ MS	F-Value	*p*-Value *	Contribution [%]
Layer height	2	122.479	61.239	58.46	<0.001	24.29
Printing orientation	2	258.463	129.232	123.37	<0.001	51.25
Printing speed	2	1.519	0.759	0.72	0.514	0.30
Layer height × printing orientation	4	108.288	27.072	25.84	<0.001	21.47
Layer height × printing speed	4	2.122	0.531	0.51	0.733	0.42
Printing orientation × printing speed	4	3.030	0.757	0.72	0.600	0.60
Error	8	8.380	1.048	-	-	1.66
Total	26	504.281	-	-	-	100

* significance level *p* ≤ 0.05.

**Table 5:** ANOVA for compression test.

Source	DF	ADJ SS	ADJ MS	F-Value	*p*-Value *	Contribution [%]
Layer height	2	31.38	15.688	3.88	0.066	2.42
Printing orientation	2	1141.76	570.879	141.28	<0.001	88
Printing speed	2	47.37	23.683	5.86	0.027	3.65
Layer height × printing orientation	4	7.33	1.832	0.45	0.768	0.56
Layer height × printing speed	4	17.84	4.460	1.10	0.418	1.38
Printing orientation × Printing speed	4	19.41	4.853	1.20	0.381	1.50
Error	8	32.33	4.041	-	-	2.49
Total	26	1297.41	-	-	-	100

* significance level *p* ≤ 0.05.

**Table 6:** GRA results.

Experiment Number	Tensile Coefficient	Compression Coefficient	GRA Score
1	0.94	0.92	0.93
2	0.94	1.00 ^H^	0.97
3	1.00 ^H^	0.66	0.83
4	0.51	0.42	0.47
5	0.44	0.42	0.43
6	0.42	0.39	0.40
7	0.37	0.41	0.39
8	0.40	0.41	0.40
9	0.41	0.40	0.40
10	0.58	0.99	0.79
11	0.64	0.86	0.75
12	0.72	0.50	0.61
13	0.39	0.37	0.38
14	0.40	0.38	0.39
15	0.41	0.38	0.39
16	0.39	0.44	0.41
17	0.41	0.38	0.40
18	0.40	0.34	0.37
19	0.40	0.74	0.57
20	0.41	0.81	0.61
21	0.42	0.75	0.59
22	0.33 ^L^	0.37	0.35
23	0.36	0.36	0.36
24	0.34	0.37	0.36
25	0.42	0.35	0.38
26	0.39	0.40	0.39
27	0.42	0.33 ^L^	0.37

^H^ Highest value for compression and tensile strength; ^L^ lowest value for compression and tensile strength.

## Data Availability

Data are available and can be provided upon request.

## Abbreviations

The following abbreviations are used in this manuscript:

PLA	Polylactic acid
aPHA	Polylactic acid/amorphous
PHA	Polyhydroxyalkanoate
CAD	Computer aided design
ANOVA	Analysis of variance
GRA	Grey relational analysis
GRC	Grey relational coefficient
ASTM	American society for testing and materials
H	High
L	Low
DF	Degree of freedom
ADJ SS	Adjusted sums of squares
ADJ MS	Adjusted mean squares
